# Mortality prediction model for the triage of COVID-19, pneumonia, and mechanically ventilated ICU patients: A retrospective study

**DOI:** 10.1016/j.amsu.2020.09.044

**Published:** 2020-10-03

**Authors:** Logan Ryan, Carson Lam, Samson Mataraso, Angier Allen, Abigail Green-Saxena, Emily Pellegrini, Jana Hoffman, Christopher Barton, Andrea McCoy, Ritankar Das

**Affiliations:** a Dascena, Inc., San Francisco, CA, USA; b Cape Regional Medical Center, Cape May Court House, NJ, USA

**Keywords:** Mortality prediction, COVID-19, SARS-CoV-2, Machine learning, Artificial intelligence

## Abstract

**Rationale:**

Prediction of patients at risk for mortality can help triage patients and assist in resource allocation.

**Objectives:**

Develop and evaluate a machine learning-based algorithm which accurately predicts mortality in COVID-19, pneumonia, and mechanically ventilated patients.

**Methods:**

Retrospective study of 53,001 total ICU patients, including 9166 patients with pneumonia and 25,895 mechanically ventilated patients, performed on the MIMIC dataset. An additional retrospective analysis was performed on a community hospital dataset containing 114 patients positive for SARS-COV-2 by PCR test. The outcome of interest was in-hospital patient mortality.

**Results:**

When trained and tested on the MIMIC dataset, the XGBoost predictor obtained area under the receiver operating characteristic (AUROC) values of 0.82, 0.81, 0.77, and 0.75 for mortality prediction on mechanically ventilated patients at 12-, 24-, 48-, and 72- hour windows, respectively, and AUROCs of 0.87, 0.78, 0.77, and 0.734 for mortality prediction on pneumonia patients at 12-, 24-, 48-, and 72- hour windows, respectively. The predictor outperformed the qSOFA, MEWS and CURB-65 risk scores at all prediction windows. When tested on the community hospital dataset, the predictor obtained AUROCs of 0.91, 0.90, 0.86, and 0.87 for mortality prediction on COVID-19 patients at 12-, 24-, 48-, and 72- hour windows, respectively, outperforming the qSOFA, MEWS and CURB-65 risk scores at all prediction windows.

**Conclusions:**

This machine learning-based algorithm is a useful predictive tool for anticipating patient mortality at clinically useful timepoints, and is capable of accurate mortality prediction for mechanically ventilated patients as well as those diagnosed with pneumonia and COVID-19.

## Highlights

• Mortality predictions have not previously been evaluated for COVID-19 patients.
• Machine learning may be a useful predictive tool for anticipating patient mortality.
• Prediction can be estimated at clinically useful windows up to 72 h in advance.

## 1 Introduction

Infection prevention and control recommendations from the World Health Organization (WHO) stress that early detection, effective triage, and isolation of potentially infectious patients are essential to prevent unnecessary exposures to COVID-19 [1]. However, the rapid spread of COVID-19 has outpaced US healthcare facilities’ ability to administer diagnostic tests to guide the quarantine and triage COVID-19 patients [[2], [3], [4], [5]]. The outbreak significantly affects the availability of necessary hospital resources (i.e. respirators [6] and mechanical ventilators [[7], [8], [9], [10], [11], [12]]). COVID-19 can be lethal, with a variable case fatality rate considered to be between that of severe acute respiratory syndrome (SARS; 9.5% [13]) and influenza (0.1%) [[14], [15], [16]] and the potential to develop into severe respiratory diseases [[17], [18], [19]]. During this period of unprecedented health crisis, clinicians must prioritize care for at-risk individuals to maximize limited resources. Mortality prediction tools aid in triage and resource allocation by providing advance warning of patient deterioration. Our prior work has validated machine-learning (ML) algorithms for their ability to predict mortality and patient stability in a variety of settings and on diverse patient populations [[20], [21], [22], [23], [24]].

## 2 Theory

Of particular interest during the COVID-19 pandemic is mortality prediction of COVID-19 patients, as well as those who have developed respiratory complications such as pneumonia and conditions requiring mechanical ventilation. Some prior studies predicting mortality in the mechanically vented subpopulation have used a logistic regression model. When applied on day 21 [25] or 14 [26,27] of mechanical ventilation, this provides a probability of 1-year mortality. These studies were designed to determine the long-term prognosis of patients receiving prolonged mechanical ventilation. Here we present a mortality prediction tool applied to intensive care unit (ICU) patients requiring mechanical ventilation as well as those diagnosed with pneumonia, with mortality prediction windows of 12, 24, 48 and 72 h prior to death. We apply this algorithm for the same mortality prediction windows in COVID-19 patients.

## 3 Materials and methods

### 3.1 Data sources

Patient records were collected from the Medical Information Mart for Intensive Care (MIMIC) dataset, an openly available dataset developed by the MIT Lab for Computational Physiology, comprising de-identified health data associated with ~60,000 intensive care unit admissions [28]. It includes demographics, vital signs, laboratory tests, medications, and more. Data collection was passive with no impact on patient safety. MIMIC data has been de-identified in compliance with the Health Insurance Portability and Accountability Act (HIPAA) Privacy Rule.

Patient records of COVID-19 polymerase chain reaction (PCR) positive patients were collected from a community hospital and formatted in the same manner as the MIMIC dataset. A total of 114 patient encounters were collected between 12 March and 12 April 2020. Data collection was passive with no impact on patient safety. Dascena establishes de-identification by removing all protected health information (PHI) identifiers and by jittering all timestamps (including date of birth (DOB)) randomly either forwards or backwards in time. Studies performed on de-identified patient data constitute non-human subjects research, and thus this study has been determined by the Pearl Institutional Review Board to be Exempt according to FDA 21 CFR 56.104 and 45CFR46.104(b) (4): (4) Secondary Research Uses of Data or Specimens under study number 20-DASC-119.

#### 3.1.1 Data processing

For the MIMIC and community hospital datasets, we included only records for patients aged 18 years or older. We excluded patient records for which there were no raw data or no discharge or death dates. We then filtered for length of stay (LOS) for the different look aheads of 12, 24, 48, and 72 h. Table 1 lists the number of patients for each inclusion criterion from the MIMIC dataset. Inclusion criteria for the community hospital dataset are listed in Table 2. We minimally processed raw electronic health record (EHR) data to generate features. Following imputation of missing values, we averaged one value for each measurement each hour for up to 3 h preceding prediction time. We also calculated differences between the current hour and the prior hour and between the prior hour and the hour before that. We concatenated these values from each measurement into a feature vector. For the MIMIC dataset, pneumonia patients were identified by International Classification of Diseases (ICD) codes, while those requiring mechanical ventilation and their corresponding start times were determined by chart measurements indicative of a mechanical ventilation setting. In the community hospital dataset, COVID-19 patients were identified with positive SARS-Cov2 PCR tests.

**Table 1:** Inclusion criteria for patients in the MIMIC dataset. *Required measurements include Age, Heart Rate, Respiratory Rate, Peripheral Oxygen Saturation (SpO2), Temperature, Systolic Blood Pressure, Diastolic Blood Pressure, White Blood Cell Counts, Platelets, Lactate, Creatinine, and Bilirubin.

Criterion	Encounters
ICU stays in MIMIC	61,532
ICU stays with patients aged ≥ 18 years, any measurements present*	53,001
Length of stay filtering for all patients 12 h	50,695
Length of stay filtering for all patients 24 h	40,959
Length of stay filtering for all patients 48 h	26,576
Length of stay filtering for all patients 72 h	18,275
Length of stay filtering for mechanically ventilated patients 12 h	24,934
Length of stay filtering for mechanically ventilated patients 24 h	21,414
Length of stay filtering for mechanically ventilated patients 48 h	16,085
Length of stay filtering for mechanically ventilated patients 72 h	12,368
Length of stay filtering for pneumonia patients 12 h	8879
Length of stay filtering for pneumonia patients 24 h	7678
Length of stay filtering for pneumonia patients 48 h	5600
Length of stay filtering for pneumonia patients 72 h	4169

**Table 2:** Inclusion criteria for patients in the community hospital dataset. *Required measurements include Age, Heart Rate, Respiratory Rate, Peripheral Oxygen Saturation (SpO2), Temperature, Systolic Blood Pressure, Diastolic Blood Pressure, White Blood Cell Counts, Platelets, Lactate, Creatinine, and Bilirubin.

Criterion	Encounters
COVID positive stays in community hospital	114
COVID positive stays with patients aged ≥ 18 years, any measurements present*	114
Length of stay filtering for all COVID positive patients 12 h	114
Length of stay filtering for all COVID positive patients 24 h	112
Length of stay filtering for all COVID positive patients 48 h	110
Length of stay filtering for all COVID positive patients 72 h	103

Data were discretized into 1 h intervals, beginning at the time of the first recorded patient measurement and hourly measurements were required for each input variable. Measurements were averaged to produce a single value in cases when multiple observations of the same patient measurement were taken within a given hour. This ensures that the measurement rate was the same across patients and across time. Missing values were imputed by carrying forward the most recent past measurement in cases where no measurement of a clinical variable was available for a given hour. For some patients with infrequent measurements of one or more vital signs, this simple imputation resulted in many consecutive hours with identical values.

Our publication on the use of gradient boosted trees for sepsis detection and prediction describes the data processing in detail [29]. Predictions were generated for all experiments using the following variables: Age, Heart Rate, Respiratory Rate, Peripheral Oxygen Saturation (SpO2), Temperature, Systolic Blood Pressure, Diastolic Blood Pressure, White Blood Cell Counts, Platelets, Lactate, Creatinine, and Bilirubin, over an interval of 3 h and their corresponding differentials in that interval.

### 3.2 Gold standard

The outcome of interest was in-hospital patient mortality, determined retrospectively for each patient. In the MIMIC dataset, we used the expire_flag field to identify the last stays of those patients. Similarly, the community hospital dataset contains a deceased flag that is either true or false to determine mortality.

#### 3.2.1 The machine learning algorithm

The classifier was created using the XGBoost method for fitting “boosted” decision trees. We applied the XGBoost package for Python32 to the patient age and vital sign measurements and their temporal changes, where temporal changes included hourly differences between each measurement beginning 3 h before prediction time. Gradient boosting, which XGBoost implements, is an ensemble learning technique that combines results from multiple decision trees to create prediction scores. Each tree splits the patient population into smaller and smaller groups, successively. Each branch splits the patients who enter it into two groups, based on whether their value of some covariate is above or below some threshold—for instance, a branch might divide patients according to whether their temperature is above or below 100 °F. After some number of branches, the tree ends in a set of “leaves.” Each patient is in exactly one leaf, according to the values of his or her measurements. Each “leaf” of the tree is predicted to have the same risk of mortality. The covariate involved in each split and the threshold value are selected by an algorithm designed to trade off fit to the training data and accuracy on out-of-sample data by using cross-validation to avoid “over-fitting.” We restricted tree depth to a maximum of six branching levels, set the learning rate parameter of XGBoost to 0.1, and restricted the tree ensembles to 1000 trees to limit the computational burden.

Hyperparameter optimization was performed using cross-validated grid search. We included a hyperparameter for the early stopping of the iterative tree-addition procedure to prevent overfit of the model on the training data and optimized across this hyperparameter using fivefold cross-validation. Due to computational and time constraints, hyperparameter optimization was performed across a sparse parameter grid, where the candidate hyperparameter values were chosen to span large ranges of viable parameter space. Cross-validated grid search was conducted to determine the optimal combination of candidate hyperparameters. While XGBoost has a large number of trainable parameters, computational and time constraints limited the set of parameters to be tuned to just those parameters with the largest impact on performance on the training data and most relevant to the prediction task.

To validate the boosted tree predictor when training and testing was performed on data from the same institution, we used fivefold cross-validation. For each model, four-fifths of the patients were randomly selected to train the model and the remaining one-fifth were used as a hold-out set to test the predictions. To account for the random selection of the training set, reported performance metrics are the average performance of the five separately trained models arising from fivefold cross-validation, each of which was trained on four-fifths of the data and tested on the remaining fifth. For AUROC, we also reported the standard deviation of the five AUROC values obtained from cross-validation.

For patients who died, we modeled mortality 12, 24, 48, and 72 h before death to evaluate the performance with a variety of lead times. For mechanically ventilated encounters, the time point was the start of ventilation for positive and negative class. Predictors were trained independently for each distinct lookahead time. In 12, 24, 48 and 72 h long lookahead predictions following a 3-h window of measurements, patients must have data for, respectively, 15, 27, 51 or 75 respective hours preceding the time of in-hospital mortality or the time of discharge. Accordingly, we selected patients with the appropriate stays for the training and testing of each lookahead.

### 3.3 Comparison to rule-based methods

To calculate the AUROC for rule-based predictors of mortality, we calculated quick Sepsis Related Organ Failure Assessment (qSOFA), Modified Early Warning Score (MEWS) and CURB-65 scores for patients in the MIMIC database. qSOFA has also been used to predict poor outcomes in pneumonia patients, including the need for mechanical ventilation, and has been shown to either match or outperform other outcome predictors such as SOFA, CRB, CRB-65 and the pneumonia severity index (PSI) [30,31]. Among more generally used mortality prediction scores, qSOFA has been shown to have similar predictive performance to that of Acute Physiology, Age, Chronic Health Evaluation (APACHE) II or SOFA, as evidenced by a lack of statistical difference between AUROC [32]. The MEWS and CURB-65 scores have also been validated for mortality prediction in general patient populations [33,34] and those with community-acquired pneumonia [35] or COVID-19 [36], respectively. Scores were calculated using the entire dataset. We calculated the qSOFA score using systolic blood pressure, respiratory rate, and Glasgow Coma Scale (GCS) from EHR data. MEWS was calculated using systolic blood pressure, heart rate, respiratory rate, and temperature. GCS was used as a proxy for evaluating AVPU. CURB-65 scores were computed using age, BUN, respiratory rate, as well as systolic and diastolic blood pressure. A GCS of less than or equal to 14 was used as a proxy for confusion. Comparator score calculations for patients in the community hospital dataset were modified based on available data.

## 4 Results

XGBoost model training and testing was performed on the MIMIC dataset. Patient demographic information for all ICU encounters as well as each subpopulation are presented in Table 3, Table 4, Table 5. Patient demographic information for all encounters from the community hospital data set are listed in Table 6.

**Table 3:** Patient demographic information for MIMIC dataset for all encounters (53,001).

	Characteristic	MIMIC (%)
Age	18–29	4.70
	30–39	5.25
	40–49	10.65
	50–59	17.52
	60–69	20.99
	>70	40.90
Gender	Male	43.68
	Female	56.32
In-hospital Death	Yes	9.59
	No	90.41

**Table 4:** Patient demographic information for MIMIC dataset for all pneumonia encounters (9,166).

	Characteristic	MIMIC (%)
Age	18–29	2.97
	30–39	4.34
	40–49	9.33
	50–59	17.17
	60–69	21.01
	>70	45.18
Gender	Male	53.99
	Female	46.01
In-hospital Death	Yes	11.41
	No	88.59

**Table 5:** Patient demographic information for MIMIC dataset for all mechanically ventilated encounters (25,895).

	Characteristic	MIMIC (%)
Age	18–29	4.50
	30–39	4.38
	40–49	10.16
	50–59	17.91
	60–69	22.39
	>70	40.66
Gender	Male	59.1
	Female	40.9
In-hospital Death	Yes	15.73
	No	84.27

**Table 6:** Patient demographic information for community hospital dataset encounters.

	Characteristic	Community Hospital (%)
Age	18–29	7.00
	30–39	10.5
	40–49	8.77
	50–59	15.79
	60–69	23.68
	>70	34.21
Gender	Male	58.77
	Female	41.33
In-hospital Death	Yes	21.0
	No	79.0

**Fig. 1:**
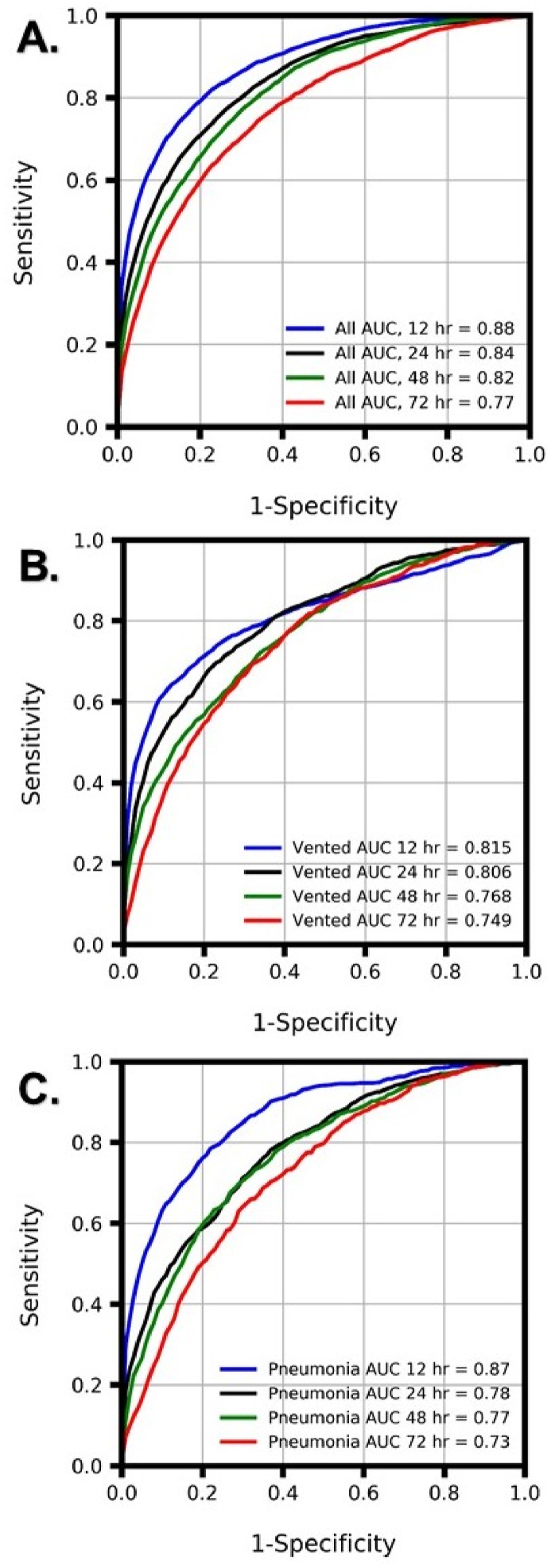
Comparison of area under the receiver operating characteristic (AUROC) curves for XGBoost models. AUROCs for the boosted tree predictor are presented for 12-, 24-, 48-, and 72-h mortality prediction with training and testing performed on MIMIC data from (A) all ICU patients as well as subpopulations of (B) mechanically ventilated (vented) ICU patients and (C) pneumonia ICU patients.

The XGBoost ML algorithm predicted mortality in all ICU patients as well as mechanically ventilated and pneumonia patients more accurately than qSOFA, MEWS and CURB-65 at all prediction windows ( Table 7, Table 8 and Supplementary Table S5). When trained and tested on the MIMIC dataset, the XGBoost predictor obtained AUROCs of 0.82, 0.81, 0.77, and 0.75 for mortality prediction on mechanically ventilated patients at 12-, 24-, 48-, and 72- hour windows, respectively, and AUROCs of 0.87, 0.78, 0.77, and 0.73 for mortality prediction on pneumonia patients at 12-, 24-, 48-, and 72- hour windows, respectively (Fig. 1). Feature importance statistics are listed in Table S1, Table S2, Table S3, Table S4.

**Table 7:** Comparison of AUROC, average precision (APR), sensitivity, specificity, F1, diagnostic odds ratio (DOR), positive and negative likelihood ratios (LR+ and LR‒), accuracy and recall obtained by the machine learning algorithm (MLA) and the qSOFA score for mortality prediction at 12-, 24-, 48-, and 72- hour windows on pneumonia patients using the MIMIC dataset. Standard deviations are listed in parenthesis. For AUROC and APR the operating point was set near a sensitivity of 0.800.

		MLA: Pneumonia	qSOFA: Pneumonia	MEWS: Pneumonia	CURB-65: Pneumonia
12 h	AUROC	0.865 (0.0027)	0.719	0.792	0.595
	APR	0.594 (0.0075)	0.242	0.405	0.154
	Sensitivity	0.800 (0.0000)	0.933	0.884	0.973
	Specificity	0.761 (0.0101)	0.304	0.472	0.169
	F1	0.467 (0.0094)	0.280	0.323	0.255
	DOR	12.74 (0.715)	6.126	6.831	7.397
	LR+	3.35 (0.143)	1.342	1.674	1.171
	LR-	3.35 (0.143)	1.342	1.674	1.171
	Accuracy	0.766 (0.0088)	0.385	0.524	0.272
	Recall	0.804 (0.0000)	0.933	0.884	0.973
24 h	AUROC	0.783 (0.0017)	0.721	0.779	0.612
	APR	0.442 (0.0070)	0.267	0.346	0.159
	Sensitivity	0.802 (0.0000)	0.932	0.906	0.974
	Specificity	0.594 (0.0324)	0.285	0.439	0.142
	F1	0.349 (0.0160)	0.271	0.313	0.247
	DOR	5.99 (0.727)	5.484	7.552	6.211
	LR+	1.99 (0.144)	1.304	1.614	1.136
	LR-	1.99 (0.144)	1.304	1.614	1.136
	Accuracy	0.621 (0.0283)	0.367	0.498	0.248
	Recall	0.802 (0.0000)	0.932	0.906	0.974
48 h	AUROC	0.769 (0.0074)	0.681	0.747	0.606
	APR	0.407 (0.0099)	0.264	0.334	0.178
	Sensitivity	0.803 (0.0000)	0.917	0.866	0.975
	Specificity	0.580 (0.0308)	0.238	0.394	0.122
	F1	0.374 (0.0158)	0.284	0.317	0.271
	DOR	5.67 (0.701)	3.454	4.211	5.318
	LR+	1.92 (0.138)	1.203	1.429	1.110
	LR-	1.92 (0.138)	1.203	1.429	1.110
	Accuracy	0.612 (0.0264)	0.335	0.462	0.245
	Recall	0.803 (0.0000)	0.917	0.866	0.975
72 h	AUROC	0.726 (0.0047)	0.645	0.668	0.592
	APR	0.333 (0.0168)	0.227	0.275	0.185
	Sensitivity	0.801 (0.0030)	0.933	0.867	0.970
	Specificity	0.507 (0.0137)	0.202	0.333	0.098
	F1	0.357 (0.0070)	0.296	0.315	0.281
	DOR	4.16 (0.307)	3.542	3.250	3.541
	LR+	1.63 (0.052)	1.169	1.300	1.075
	LR-	1.63 (0.052)	1.169	1.300	1.075
	Accuracy	0.553 (0.0120)	0.315	0.416	0.233
	Recall	0.807 (0.0000)	0.933	0.867	0.970

**Table 8:** Comparison of AUROC, average precision (APR), sensitivity, specificity, F1, diagnostic odds ratio (DOR), positive and negative likelihood ratios (LR+ and LR‒), accuracy and recall obtained by the machine learning algorithm (MLA) and the qSOFA score for mortality prediction at 12-, 24-, 48-, and 72- hour windows on mechanically ventilated patients using the MIMIC dataset. Standard deviations are listed in parenthesis. For AUROC and APR the operating point was set near a sensitivity of 0.800.

		MLA: Mechanically Ventilated	qSOFA: Mechanically Ventilated	MEWS: Mechanically Ventilated	CURB-65: Mechanically Ventilated
12 h	AUROC	0.815 (0.0030)	0.731	0.808	0.620
	APR	0.598 (0.0055)	0.276	0.417	0.173
	Sensitivity	0.803 (0.0016)	0.969	0.845	0.988
	Specificity	0.647 (0.0241)	0.232	0.630	0.098
	F1	0.394 (0.0159)	0.280	0.400	0.253
	DOR	7.54 (0.902)	9.414	9.287	9.237
	LR+	2.29 (0.167)	1.261	2.285	1.096
	LR-	2.29 (0.167)	1.261	2.285	1.096
	Accuracy	0.668 (0.0211)	0.331	0.659	0.218
	Recall	0.802 (0.0000)	0.969	0.845	0.988
24 h	AUROC	0.806 (0.0030)	0.729	0.789	0.626
	APR	0.506 (0.0098)	0.274	0.357	0.179
	Sensitivity	0.803 (0.0017)	0.970	0.810	0.987
	Specificity	0.634 (0.0101)	0.244	0.611	0.109
	F1	0.392 (0.0060)	0.289	0.381	0.260
	DOR	7.06 (0.299)	10.384	6.714	9.343
	LR+	2.20 (0.060)	1.283	2.084	1.108
	LR-	2.20 (0.060)	1.283	2.084	1.108
	Accuracy	0.658 (0.0087)	0.344	0.638	0.230
	Recall	0.802 (0.0000)	0.970	0.810	0.987
48 h	AUROC	0.768 (0.0034)	0.715	0.753	0.611
	APR	0.488 (0.0048)	0.312	0.357	0.209
	Sensitivity	0.804 (0.0019)	0.977	0.826	0.977
	Specificity	0.553 (0.0091)	0.182	0.546	0.085
	F1	0.398 (0.0046)	0.322	0.403	0.298
	DOR	5.08 (0.198)	9.266	5.710	3.840
	LR+	1.80 (0.038)	1.194	1.818	1.067
	LR-	1.80 (0.038)	1.194	1.818	1.067
	Accuracy	0.595 (0.0076)	0.315	0.592	0.233
	Recall	0.803 (0.0000)	0.977	0.826	0.977
72 h	AUROC	0.749 (0.0053)	0.657	0.665	0.601
	APR	0.406 (0.0115)	0.261	0.278	0.213
	Sensitivity	0.805 (0.0000)	0.977	0.943	0.983
	Specificity	0.558 (0.0135)	0.153	0.261	0.070
	F1	0.413 (0.0068)	0.327	0.347	0.308
	DOR	5.22 (0.288)	7.683	5.799	4.322
	LR+	1.82 (0.056)	1.154	1.276	1.057
	LR-	1.82 (0.056)	1.154	1.276	1.057
	Accuracy	0.601 (0.0111)	0.297	0.380	0.230
	Recall	0.805 (0.0000)	0.977	0.943	0.983

Detailed performance metrics for the XGBoost predictor on pneumonia and mechanically ventilated patients are presented in Table 7, Table 8 and on COVID-19 patients in Table 9. All predictor training and testing was performed on the MIMIC data set. The diagnostic odds ratio (DOR) is a measure for comparing diagnostic accuracy between tools and is calculated as (True Positive/False Negative)/(False Positive/True Negative). DOR represents the ratio of the odds of a true positive prediction of mortality in patients who died within a certain prediction window to the odds of a false positive prediction of mortality in patients who did not die within a certain prediction window. For all prediction windows, the XGBoost predictor had a higher DOR than qSOFA.

**Table 9:** Comparison of AUROC, average precision (APR), sensitivity, specificity, F1, diagnostic odds ratio (DOR), positive and negative likelihood ratios (LR+ and LR‒), accuracy and recall obtained by the ML algorithm (MLA) and the qSOFA, MEWS and CURB-65 scores for mortality prediction at 12-, 24-, 48-, and 72- hour windows on 114 COVID-19 PCR Positive Patients from the community hospital data set. Standard deviations are listed in parenthesis. For AUROC and APR the operating point was set near a sensitivity of 0.800. n/a (not applicable).

		MLA	qSOFA	MEWS	CURB-65
12 h	AUROC	0.910 (0.0024)	0.791	0.769	0.780
	APR	0.795 (0.0054)	0.510	0.514	0.369
	Sensitivity	0.826 (0.0000)	1.000	1.000	1.000
	Specificity	0.804 (0.0239)	0.000	0.022	0.500
	F1	0.638 (0.0228)	0.338	0.343	0.505
	DOR	19.89 (2.912)	n/a	n/a	n/a
	LR+	4.29 (0.506)	1.000	1.023	2.000
	LR-	4.29 (0.506)	1.000	1.023	2.000
	Accuracy	0.809 (0.0191)	0.204	0.221	0.602
	Recall	0.826 (0.0000)	1.000	1.000	1.000
24 h	AUROC	0.903 (0.0059)	0.840	0.780	0.764
	APR	0.754 (0.0127)	0.563	0.515	0.354
	Sensitivity	0.826 (0.0000)	0.826	1.000	1.000
	Specificity	0.816 (0.0054)	0.822	0.033	0.444
	F1	0.649 (0.0054)	0.655	0.346	0.479
	DOR	21.03 (0.770)	21.969	n/a	n/a
	LR+	4.48 (0.134)	4.647	1.034	1.800
	LR-	4.48 (0.134)	4.647	1.034	1.800
	Accuracy	0.818 (0.0043)	0.823	0.230	0.558
	Recall	0.826 (0.0000)	0.826	1.000	1.000
48 h	AUROC	0.862 (0.0088)	0.792	0.724	0.802
	APR	0.684 (0.0156)	0.478	0.444	0.384
	Sensitivity	0.818 (0.0000)	1.000	0.955	1.000
	Specificity	0.773 (0.0334)	0.000	0.022	0.522
	F1	0.598 (0.0297)	0.328	0.321	0.506
	DOR	15.80 (3.051)	n/a	0.477	n/a
	LR+	3.69 (0.555)	1.000	0.976	2.093
	LR-	3.69 (0.555)	1.000	0.976	2.093
	Accuracy	0.782 (0.0268)	0.196	0.205	0.616
	Recall	0.818 (0.0000)	1.000	0.955	1.000
72 h	AUROC	0.873 (0.0034)	0.722	0.797	0.751
	APR	0.649 (0.0209)	0.364	0.452	0.320
	Sensitivity	0.819 (0.0190)	1.000	0.857	1.000
	Specificity	0.760 (0.0181)	0.000	0.611	0.467
	F1	0.576 (0.0171)	0.318	0.486	0.467
	DOR	14.64 (2.376)	n/a	9.429	n/a
	LR+	3.43 (0.261)	1.000	2.204	1.875
	LR-	3.43 (0.261)	1.000	2.204	1.875
	Accuracy	0.771 (0.0146)	0.189	0.658	0.568
	Recall	0.810 (0.0000)	1.000	0.857	1.000

These results suggest that the XGBoost predictor is capable of predicting mortality in pneumonia, mechanically ventilated, and COVID-19 patients and outperforms the qSOFA, MEWS and CURB-65 mortality risk scores.

## 5 Discussion

Accurate mortality prediction can assist with the allocation of limited hospital resources and optimize patient management. Additionally, advanced mortality prediction can facilitate decision making with family and caregivers. The commonly used MEWS [37], the APACHE [38], Simplified Acute Physiology Score (SAPS II) [39], Sepsis-Related Organ Failure Assessment (SOFA) [40], and the quick SOFA (qSOFA) score [41] provide a rough estimate of mortality prediction, however the specificity and sensitivity of these tools are limited for COVID and mechanically ventilated populations [42]. Machine learning (ML) has been previously broadly applied to predictive tasks within the biosciences [[43], [44], [45], [46]]. ML-based tools for mortality prediction have been applied to sepsis [47,48] cardiac arrest [49], coronary artery disease [50], and extubation [51] patient populations, and have been implemented in a broad range of clinical settings, including the emergency department (ED) [48] and the intensive care unit (ICU) [[52], [53], [54], [55]].

Studies of mortality prediction on pneumonia and mechanically ventilated patients are particularly relevant for COVID-19 related lung complications. We have demonstrated that machine learning algorithms are useful predictive tools for anticipating patient mortality at clinically useful windows of 12, 24, 48, and 72 h in advance and have validated mortality prediction accuracy for COVID-19, pneumonia, mechanically ventilated, and all ICU patients (Fig. 1), demonstrating that for all prediction types and windows, our ML algorithm outperforms the qSOFA, MEWS and CURB-65 severity scores (Table 7, Table 8, Table 9).

A meta-analysis of studies focusing on predicting mortality in pneumonia patients showed that of the three commonly used prognostic scores which predicted mortality, the Pneumonia Severity Index (PSI) had the highest AUROC of 0.81. However, this index was used for predicting 30-day mortality specifically among patients with community acquired pneumonia [56]. When trained and tested on the MIMIC dataset, the XGBoost predictor obtained AUROCs of 0.87, 0.78, 0.77, and 0.73 for mortality prediction on pneumonia patients at 12-, 24-, 48-, and 72- hour windows, respectively (Fig. 1, Table 7).

When trained and tested on the community hospital dataset, the XGBoost predictor obtained AUROCs of 0.91, 0.90, 0.86, and 0.87 for mortality prediction on COVID-19 PCR positive patients at 12-, 24-, 48-, and 72- hour windows, respectively (Table 9). The algorithm outperformed the qSOFA, MEWS and CURB-65 risk scores at all prediction windows (Table 9). This ML algorithm can be used to automatically monitor patient populations without incurring additional data entry or impeding clinical workflow, and patient alerts can be set to desired thresholds for sensitivity and specificity of alerting as needed in different care settings. As a clinical decision support tool, the machine learning algorithm presented in this study may assist clinicians in navigating the complexities surrounding COVID-19 related resource allocation. During a pandemic, accurate triage of patients is essential for improving patient outcomes, effectively utilizing clinical care teams, and efficiently allocating resources. The benefit of our approach is that when our machine learning algorithm is implemented in clinical ICU settings, healthcare providers can potentially identify patients at risk of significant COVID-19 related decompensation before they deteriorate, thus facilitating effective resource allocation and identifying those patients most likely to benefit from increased care.

There are several limitations to our study. The ML algorithm developed on the MIMIC dataset used only data from the ICU. Therefore, further research is required to evaluate performance of the algorithm in other patient care settings. Further, because the algorithm only utilized laboratory data and vital signs as inputs, it did not account for actions undertaken by the care team. These actions could signify aggressive treatment or withdrawal of treatment and could cause changes to algorithm inputs, potentially leading to variations in the algorithm's prediction score. On one hand, incorporating care team actions into algorithm inputs could be useful feedback to the care team in the sense that it may aid them in determining whether a given intervention was harmful or beneficial. On the other hand, accounting for actions undertaken by the care team may complicate the interpretation of what it means to “anticipate” mortality, given that the current state of knowledge of the care team is unknown. Finally, because this is a retrospective study, we cannot determine the performance of the mortality prediction algorithm in a prospective clinical setting. Prospective validation is required to determine how clinicians may respond to risk predictions as well as whether predictions can affect patient outcomes or resource allocation.

## 6 Conclusion

The ML algorithm presented in this study is a useful predictive tool for anticipating patient mortality at clinically useful windows up to 72 h in advance, and capable of accurate mortality prediction for COVID-19, pneumonia, and mechanically ventilated patients.

## Patient and public involvement statement

Patients and the public were not involved in the design and conduct of the study, choice of outcome measures, or recruitment to the study due to the nature of data collection.

## Dissemination declaration

**Transparency declaration:** RD affirms that the manuscript is an honest, accurate, and transparent account of the study being reported; that no important aspects of the study have been omitted; and that any discrepancies from the study as originally planned (and, if relevant, registered) have been explained.

## Funding sources

N/A.

## Ethical approval

Data has been deidentified and, as such, does not constitute human subjects research.

## Conflicts of interest

All authors who have affiliations listed with Dascena (San Francisco, California, USA) are employees or contractors of Dascena.

## Trial registration

This study has been registered on ClinicalTrials.gov under study number NCT04358510.

## Provenance and peer review

Not commissioned, externally peer reviewed.

## Guarantor

The Guarantor is the one or more people who accept full responsibility for the work and/or the conduct of the study, had access to the data, and controlled the decision to publish

## CRediT authorship contribution statement

**Logan Ryan:** Methodology, Investigation. **Carson Lam:** Conceptualization, Methodology, Formal analysis, Writing - original draft, Supervision. **Samson Mataraso:** Conceptualization, Methodology, Formal analysis, Writing - original draft, Supervision. **Angier Allen:** Investigation, Writing - original draft. **Abigail Green-Saxena:** Writing - original draft. **Emily Pellegrini:** Writing - original draft. **Jana Hoffman:** Conceptualization, Methodology, Formal analysis, Writing--Original Draft, Supervision. **Christopher Barton:** Supervision. **Ritankar Das:** had full access to all of the data in the study and takes responsibility for the integrity of the data and the accuracy of the data analysis, Conceptualization, Methodology, Formal analysis, Writing - original draft, Supervision.

## Figures and Tables

**Table S1:** Feature Importance 12 h outlook using MIMIC dataset

Rank	Feature	Importance (f_score)
1	SpO2_-2	81
2	HR	77
3	SysABP_diff_-2	76
4	DiasABP_-0	68
5	SysABP_-0	68
6	WBC_-1	66
7	HR_-2	64
8	Platelets_-1	64
9	RespRate_diff_-1	63
10	SpO2_diff_-1	62

**Table S2:** Feature Importance 24 h outlook using MIMIC dataset

Rank	Feature	Importance (f_score)
1	SpO2_diff_-1	92
2	DiasABP_-1	69
3	Platelets_-1	68
4	DiasABP_diff_-1	64
5	Temp_-1	61
6	RespRate_-2	60
7	DiasABP_diff_-2	60
8	WBC_-1	60
9	HR_diff_-1	57
10	RespRate_diff_-1	56

**Table S3:** Feature Importance 48 h outlook using MIMIC dataset

Rank	Feature	Importance (f_score)
1	WBC_-2	48
2	DiasABP_diff_-2	44
3	HR_-2	43
4	DiasABP_-1	41
5	RespRate_-1	41
6	Temp_diff_-2	38
7	Temp_diff_-1	36
8	Temp_-0	35
9	SpO2_diff_-2	35
10	WBC_-1	34

**Table S4:** Feature Importance 72 h outlook using MIMIC dataset

Rank	Feature	Importance (f_score)
1	SysABP_diff_-1	61
2	Platelets_-0	46
3	SpO2_-0	46
4	DiasABP_-2	45
5	Lactate_-0	45
6	RespRate_-0	45
7	SysABP_-1	44
8	Creatinine_-1	42
9	Temp_-2	42
10	RespRate_diff_-1	37

**Table S5:** Comparison of AUROC, average precision (APR), sensitivity, specificity, F1, diagnostic odds ratio (DOR), positive and negative likelihood ratios (LR+ and LR‒), accuracy and recall obtained by the machine learning algorithm (MLA) and the qSOFA score for mortality prediction at 12-, 24-, 48-, and 72- hour windows on all ICU patients using the MIMIC dataset. Standard deviations are listed in parenthesis. For AUROC and APR the operating point was set near a sensitivity of 0.800.

		MLA: All ICU	qSOFA: All ICU	MEWS: All ICU	CURB-65: All ICU
12 h	AUROC	0.862 (0.0012)	0.760	0.833	0.652
	APR	0.553 (0.0018)	0.225	0.392	0.131
	Sensitivity	0.801 (0.0000)	0.949	0.897	0.984
	Specificity	0.750 (0.0046)	0.373	0.559	0.185
	F1	0.378 (0.0040)	0.236	0.290	0.198
	DOR	12.09 (0.297)	11.085	11.054	14.088
	LR+	3.21 (0.059)	1.513	2.033	1.208
	LR-	3.21 (0.059)	1.513	2.033	1.208
	Accuracy	0.755 (0.0041)	0.426	0.590	0.260
	Recall	0.801 (0.0000)	0.949	0.897	0.984
24 h	AUROC	0.819 (0.0018)	0.742	0.804	0.636
	APR	0.432 (0.0052)	0.223	0.339	0.136
	Sensitivity	0.800 (0.0000)	0.939	0.896	0.978
	Specificity	0.671 (0.0036)	0.357	0.524	0.178
	F1	0.338 (0.0023)	0.245	0.292	0.210
	DOR	8.18 (0.131)	8.629	9.473	9.692
	LR+	2.43 (0.026)	1.462	1.882	1.189
	LR-	2.43 (0.026)	1.462	1.882	1.189
	Accuracy	0.684 (0.0032)	0.416	0.562	0.258
	Recall	0.800 (0.0000)	0.939	0.896	0.978
48 h	AUROC	0.789 (0.0016)	0.706	0.760	0.616
	APR	0.408 (0.0024)	0.232	0.297	0.158
	Sensitivity	0.801 (0.0000)	0.945	0.882	0.977
	Specificity	0.619 (0.0110)	0.282	0.437	0.137
	F1	0.356 (0.0063)	0.269	0.301	0.242
	DOR	6.55 (0.315)	6.781	5.771	6.791
	LR+	2.10 (0.063)	1.316	1.565	1.132
	LR-	2.10 (0.063)	1.316	1.565	1.132
	Accuracy	0.641 (0.0097)	0.364	0.492	0.241
	Recall	0.801 (0.0000)	0.945	0.882	0.977
72 h	AUROC	0.746 (0.0026)	0.655	0.685	0.603
	APR	0.356 (0.0017)	0.218	0.256	0.182
	Sensitivity	0.802 (0.0015)	0.929	0.872	0.963
	Specificity	0.546 (0.0049)	0.247	0.388	0.111
	F1	0.361 (0.0025)	0.295	0.321	0.270
	DOR	4.86 (0.114)	4.299	4.296	3.216
	LR+	1.77 (0.020)	1.233	1.423	1.083
	LR-	1.77 (0.020)	1.233	1.423	1.083
	Accuracy	0.583 (0.0043)	0.347	0.459	0.236
	Recall	0.801 (0.0000)	0.929	0.872	0.963
